# Egr-1 is a critical regulator of EGF-receptor-mediated expansion of subventricular zone neural stem cells and progenitors during recovery from hypoxia–hypoglycemia

**DOI:** 10.1042/AN20120032

**Published:** 2013-07-11

**Authors:** Dhivyaa Alagappan, Murugabaskar Balan, Yuhui Jiang, Rachel B. Cohen, Sergei V. Kotenko, Steven W. Levison

**Affiliations:** *Department of Neurology and Neuroscience, University Hospital Cancer Center, UMDNJ-New Jersey Medical School, Newark, NJ 07103, U.S.A.; †Department of Biochemistry and Molecular Biology, University Hospital Cancer Center, UMDNJ-New Jersey Medical School, Newark, NJ 07103, U.S.A.

**Keywords:** brain injury, cell proliferation, epidermal growth factor receptor, hypoglycemia, stress, transcription factors, CNS, central nervous system, DAPI, 4′,6-diamidino-2-phenylindole, DIV, days *in vitro*, EGF-R, epidermal growth factor receptor, NF-κB, nuclear factor kappa B, NP, neural precursor, REST, relative expression software tool, SiRNAs, small interfering RNA, SVZ, subventricular zone

## Abstract

We recently established that the EGF-R (epidermal growth factor receptor) (EGF-R) is an essential regulator of the reactive expansion of SVZ (subventricular zone) NPs (neural precursors) that occurs during recovery from hypoxic-ischemic brain injury. The purpose of the current studies was to identify the conditions and the transcription factor (s) responsible for inducing the EGF-R. Here, we show that the increase in EGF-R expression and the more rapid division of the NPs can be recapitulated in *in vitro* by exposing SVZ NPs to hypoxia and hypoglycemia simultaneously, but not separately. The EGF-R promoter has binding sites for multiple transcription factors that includes the zinc finger transcription factor, Egr-1. We show that Egr-1 expression increases in NPs, but not astrocytes, following hypoxia and hypoglycemia where it accumulates in the nucleus. To determine whether Egr-1 is necessary for EGF-R expression, we used SiRNAs (small interfering RNA) specific for Egr-1 to decrease Egr-1 expression. Knocking-down Egr-1 decreased basal levels of EGF-R and it abolished the stress-induced increase in EGF-R expression. By contrast, HIF-1 accumulation did not contribute to EGF-R expression and FGF-2 only modestly induced EGF-R. These studies establish a new role for Egr-1 in regulating the expression of the mitogenic EGF-R. They also provide new information into mechanisms that promote NP expansion and provide insights into strategies for amplifying the numbers of stem cells for CNS (central nervous system) regeneration.

## INTRODUCTION

The SVZ (subventricular zone) is the largest reservoir of somatic stem cells in the brain and studies using a variety of injury models have shown that these precursors transiently expand in response to CNS (central nervous system) injuries (Szele and Chesselet, 1996; Arvidsson et al., 2002; Plane et al., 2004; Felling et al., 2006; Yang and Levison, 2006). Using the Vannucci model of neonatal hypoxic-ischemic brain injury in rats, we previously established that there is an increase in proliferating cells within the first few days after injury that results in a doubling of tripotential stem/progenitor cells within the SVZ (Felling et al., 2006). This expansion of SVZ precursors precedes the production of new neocortical and striatal neurons (Yang and Levison, 2006; Yang et al., 2007; Yang and Levison, 2007). While the SVZ expands we do not fully understand the mechanisms that mediate this increase. More recently we have established that the EGF-R (epidermal growth factor receptor) is an essential regulator of the reactive expansion of SVZ NPs (neural precursors). EGF-R acquisition recruited quiescent SVZ cells to proliferate and it increased their rate of proliferation (Alagappan et al., 2009).

EGF-R is a 170 kDa membrane spanning glycoprotein receptor tyrosine kinase that is essential for the normal development of many organs (Kornblum et al., 1998; Sibilia et al., 1998). In the CNS, the early precursors of the ventricular zone are initially FGF responsive, but as a consequence of FGF-R stimulation they begin to express the EGF-R (Ciccolini and Svendsen, 1998; Lillien and Raphael, 2000). These EGF-R expressing NPs seed the SVZ, during fetal development, giving rise to different neural cell types than the ventricular zone cells, and they persist in the SVZ as the so-called ‘adult’ neural stem cells.

The EGF-R promoter is a GC-rich TATA-less region that contains binding sites for many transcription factors that include Egr-1,WT1, TCC binding factors, AP-1, AP-2 and p53 (Ishii et al., 1985; Johnson et al., 1988; Hudson et al., 1990). EGF-R expression has been shown to be sensitive to many types of stresses such as hypoxia, UV radiation and ionizing radiation (Laderoute et al., 1992; Sachsenmaier et al., 1994; Schmidt-Ullrich, 1994). However, it has not been established which transcription factor (s) induces EGF-R expression on SVZ cells. Understanding how EGF-R expression is induced in the brain after injury will provide new insights towards developing therapeutics to enhance brain repair.

Hypoxia induces several stress response genes such as Ras, HIF-1α (hypoxia-inducible factor 1α), NF-κB (nuclear factor kappa B), Src, p53, AP-1 and Egr-1 (Wang and Semenza, 1993; Graeber et al., 1994; Koong et al., 1994; Muller et al., 1997; Yan et al., 1999). Egr-1, (also known as Krox-20, *zif* 268, TIS8, NGF1-A) encodes an 80–82 kDa zinc finger transcription factor that is a part of the multi-gene family encompassing Egr-1, Egr-3, Egr-4 and WT1 (Gashler and Sukhatme, 1995). Egr-1 binds to a GC-rich sequence, 5′-CGCCCCCGC-3′, on the promoter of several genes including EGF-R, where it can activate or repress transcription. The effects of Egr-1 are most evident during the cellular stress responses rather than under normal homoeostatic conditions. Egr-1 has been shown to regulate the expression of many genes such as tumour necrosis factor-α, transforming growth factor-β, intercellular adhesion molecule-1, macrophage colony- stimulating factor, PDGF (platelet-derived growth factor) A and B, NF-κB and EGF-R (Harrington et al., 1993; Khachigian et al., 1995; Liu et al., 1996; Maltzman et al., 1996; Yao et al., 1997; Nishi et al., 2002).

In this study, we sought to examine the relationship between hypoxic-ischemic stress, Egr-1, and its effect on EGF-R transcription in SVZ precursors of the neonatal brain. We show that Egr-1 accumulates in the nucleus of NPs as a direct consequence of hypoxia and hypoglycemia and that inhibiting Egr-1 expression decreases basal EGF-R levels and abolishes the stress-induced increase in EGF-R. These results provide a new target for therapeutics to promote the repair of CNS from resident stem cells.

## MATERIALS AND METHODS

### Primary and secondary neurosphere assay

The Institutional Animal Care and Use Committee of the New Jersey Medical School approved our protocols for all animal work. Wistar rat pups between days 4 and 6 were decapitated and their brains removed. Under aseptic conditions, a cut was made 2 mm from the anterior pole of the brain. A second cut was made approximately 3 mm posterior to the first cut. The hippocampus, corpus callosum and the meninges were removed under the microscope. Using forceps, 12 o’clock and 3 o’clock incisions were made and the region enclosed between the cortex and the ventricle containing the SVZ was removed and placed in fresh PGM [1 mM MgCl_2_, 0.6% (w/v) dextrose in PBS, pH 7.3]. The tissue was mechanically dissociated using forceps and then enzymatically by the addition of 0.05% trypsin/EDTA at 37°C for 7 min. The trypsin was inactivated by the addition of an equal volume of newborn calf serum. Then, the tissue was resuspended in a biochemically defined medium we denote as ‘ProN’. ProN contained DMEM (Dulbecco's modified Eagle's medium)/F12 1:1 media supplemented to 10 ng/ml d-biotin, 25 μg/ml insulin, 20 nM progesterone, 100 μM putrescine, 5ng/ml selenium, 50 μg/ml apo-transferrin, 15 mM Hepes and 50 μg/ml gentamycin. Cell culture media and supplements were purchased from either Invitrogen or Sigma. The tissue was triturated for several cycles and then the cell suspension passed through a 40 μM Nitex screen. The cells were collected by centrifugation at 200 ***g*** for 2 min and washed with ProN. The cells were counted with 0.1% Trypan Blue dye under a hemocytometer and plated at 5×10^4^ cells/ml in ProN media supplemented with 2 ng/ml EGF. The cells were cultured at 37°C in 5% (v/v) CO_2_ incubators and fed every 2 days by removing approximately half of the media and replenishing with fresh media. A neurosphere was defined as a free-floating cluster of at least 25 μm in diameter. At approximately 6 DIV (days *in vitro*), neurospheres were collected and dissociated by enzymatic digestion using Accutase (Invitrogen) and triturated to obtain single cells. Viable cells were established by Trypan Blue exclusion and plated at 5×10^4^ cells/ml in ProN media supplemented with 20 ng/ml EGF to obtain secondary neurospheres.

### *In-vitro* model of H–I

Secondary rat neurospheres were prepared as described above and plated into 6 well tissue culture plates. After 7 DIV the secondary neurospheres were exposed to hypoxia and hypoglycemia (H–H) by resuspending the spheres in EGF-supplemented (20 ng/ml) ProN that contained 3 mM glucose. The spheres were then subjected to 2% (v/v) O_2_/5% (v/v) CO_2_/balance N_2_ for 4 h in a hypoxic chamber (Biospherix). For the hypoglycemia only or hypoxia only group, the neurospheres were subjected to either 3 mM glucose-supplemented ProN media or 2% O_2_/5% CO_2_ balance N_2_ for 4 h, respectively. Control cells were maintained in EGF-supplemented ProN under normal culture conditions during this time. After the H–H interval, the neurospheres were returned to standard culture conditions.

### Astrocyte cultures

Primary mixed glial cell cultures were prepared from 1 to 2-day-old rat pups using standard methods (Levison and McCarthy, 1991). Enriched astrocyte cultures were prepared using the shaking method, whereupon the enriched astrocyte cultures were transferred to a defined medium for 5 days. This biochemically defined medium contained insulin (5 ng/ml), Progesterone (20 nM), Putrescine (100 μM), Selenium (5 ng/ml), apo-Transferrin (50 μg/ml) and penicillin/streptomycin (50 units/50 ng/ml). Astroglia cultured in this medium divided more slowly, possessed a more stellate morphology and they expressed decreased levels of Nestin (C. J. Kuhlow and S. W. Levison, unpublished work).

### RNA isolation

The neurospheres were collected by centrifugation and snap frozen in 0.5 ml Trizol (Invitrogen) in a slush of dry ice/ethanol and stored at −80°C. Just prior to extraction, the frozen neurospheres were thawed and homogenized using a hand held tissue homogenizer. Roughly 100 μl chloroform was added per sample and mixed to obtain a cloudy suspension that was centrifuged at 800 ***g*** for 5 min following which the aqueous phase was transferred to a new tube. Approximately 250 μl of 70% (v/v) ethanol was added and the mixture was applied to an RNeasy Mini-spin column (Qiagen) and the manufacturer's instructions followed to remove contaminants. The purity and amount of total RNA obtained was determined from the optical density values carried out in 96-well plates and run on using ABI Prism 7300 Sequence Detection System (Applied Biosystems). 18S RNA was used as the internal, endogenous normalization measured using a UV–VIS spectrophotometer (Becton–Dickinson). The total RNA concentration was adjusted to 200 ng/μl and stored at −80°C until further use.

### Quantitative PCR (Q-PCR)

One μg of total RNA was transcribed to cDNA using Qiagen Omniscript RT kit with random nanomers and RNaseIN (Promega). Primer pairs for the gene of interest was obtained from Taqman gene expression systems (Applied Biosystems) and the 18S primers were obtained from Lux primer design (Invitrogen, Cat # 115HM-01). Platinum-UDP supermix kit (Invitrogen) was used for reactions, which were control. Relative quantification was evaluated by the ΔΔCt method (Pfaffl et al., 2002).

### Western blotting

The neurospheres were resuspended in HES lysis buffer (10 mM Hepes, 1 mM EDTA, 250 mM Sucrose, pH 7.4) supplemented to 2 mM sodium orthovanadate. One Complete Mini Protease Inhibitor tablet (Roche Diagnostics) was added for each 10 ml buffer. The spheres were homogenized using a glass homogenizer (Kontes glass homogenizer with Pestle ‘A’). The homogenate was centrifuged at 1000 ***g*** for 10 min in a tabletop Beckman centrifuge. The supernatant was collected and protein concentration was estimated using the BCA assay (Pierce Biotech). Thirty μg of total protein was mixed with 4× NuPage LDS sample buffer and 10× NuPage reducing agent and heated for 10 min at 90°C and loaded onto 4–12% NuPage Bis–Tris pre-cast gel (Invitrogen). Approximately, 2 μl of MagicMark XP (Invitrogen) was loaded for standard molecular weight markers. Following electrophoresis, the proteins were transferred onto a nitrocellulose membrane and stained with Ponceau S to evaluate equal protein loading into the wells. The blots were blocked with 5% (w/v) non-fat dried skimmed milk powder /1% BSA in PBS–Tween followed by incubation with primary antibody in 1% BSA in PSB–Tween overnight at 4°C with gentle rocking. The primary antibodies used were rabbit polyclonal anti-EGF-R antibody (Abcam, ab15669, 1:250) or rabbit polyclonal anti-Egr-1 antibody (Santa Cruz Biotechnology, sc-20689, 1:200). Following three rinses with PBS–Tween the following day, the blots were incubated with corresponding secondary antibodies such as DAR–HRP conjugated (1:10000, Jackson ImmunoResearch) or DAM–HRP conjugated (1:5000, Jackson ImmunoResearch) for 2 h at room temperature (20°C). The blots were washed and signal developed with Western Lightning chemiluminescence reagent (PerkinElmer) as per manufacturer guidelines. The bands were visualized using a UVP EpiChem^3^ and processed with Labworks 4.0 digital quantification software (UVP).

### NP nucleofection

Nucleofections were carried out using the Rat Neural Stem Cell Nucleofector Kit according to the manufacturer's instructions (Cat # VPG 1005 Amaxa Biosystems, Gaithersburg). Briefly, 4×10^6^ NPs were mixed with 5 μg of purified plasmid DNA together with 10 μl of Rat NSC Nucleofector™ solution. The solution was then transferred to an Amaxa-certified cuvette and transfected using the Optimal Nucleofector™ program # A-33. Mock transfection controls had cells that were pulsed using the Amaxa system, but with no added DNA. After transfecting the cells with the desired plasmids they were immediately transferred to 37°C. The cells were transferred to standard culture medium after 24 h, whereupon they were used for experiments.

### Nuclear extract preparation

NPs subjected to H–H and control spheres were washed in ice-cold PBS and resuspended in 0.5 ml of ice-cold RSB buffer (100 mM Na_3_VO_4_, 1M DTT, 200 mM PMSF, 1 M Tris–HCl, 5 M NaCl, 1 M MgCl_2_) and incubated on ice for 5 min. Samples were then homogenized using 30 strokes in a hand held dounce homogenizer. The homogenate was centrifuged at 10000 ***g*** for 2 min, and the pellet was resuspended in 0.5 ml of buffer C [20 mM Hepes, 25% (v/v) glycerol, 420 mM NaCl, 100 mM Na_3_VO_4_, 1.5 mM MgCl_2_, 0.2 mM EDTA, 0.5 mM DTT, 200 mM PMSF and EDTA-free protease inhibitor mixture, pH 7.9]. Nuclei were incubated on ice for 30 min and centrifuged at 10000 ***g*** for 5 min to remove cell debris. The supernatant is the nuclear extract (Chen and Davis, 1999).

### NP immunohistochemistry

Spheres were collected and dissociated as described above to obtain viable single cells. The dissociated sphere cells were plated onto chamber slides [previously coated with 1% (w/v) poly-D-lysine] in 2% newborn calf serum in ProN media without growth factors. The cells were allowed to adhere overnight. The next day, the cells were exposed to EGF (20 ng/ml) supplemented ProN with 3 mM glucose and subjected to 2% O_2_/5% CO_2_/balance N_2_ for 4 h in a hypoxic chamber to mimic the *in vivo* hypoxic-ischemic insult that causes brain damage. Following the 4 h exposure, the cells were either fixed immediately or allowed to recover for 30 h under the standard culture conditions, whereupon they were immunostained. Cells were rinsed with PBS and fixed using 2% (v/v) paraformaldehyde for 15 min on ice. After one rinse the excess paraformaldehyde was quenched using 100 nM glycine at room temperature for 10 min. Following fixation, the cells were stained for 1 h at room temperature or overnight at 4°C with antibodies against Egr-1 (Santa Cruz Biotechnology, rabbit polyclonal 1:50) or Ki67 (Vector Laboratories, rabbit polyclonal, 1:100), respectively. The cells were thoroughly rinsed and incubated with biotin-conjugated anti-rabbit and then streptavidin conjugated Alexa 488 for Egr-1 and Rhodamine RedX conjugated anti-rabbit for Ki67 detection, respectively. The cells were rinsed. The nuclei were counterstained with DAPI (4′,6-diamidino-2-phenylindole) to identify cells and coverslipped with anti-fading Gelmount (Biomedia) and the chamber slides were allowed to dry overnight. Images of stained cells were collected using a SenSys photometrics cooled charge-coupled device camera interfaced with IP Lab Scientific imaging software (Scanalytics) on an Olympus AX-70 microscope (Olympus America).

## RESULTS

**Figure 1:**
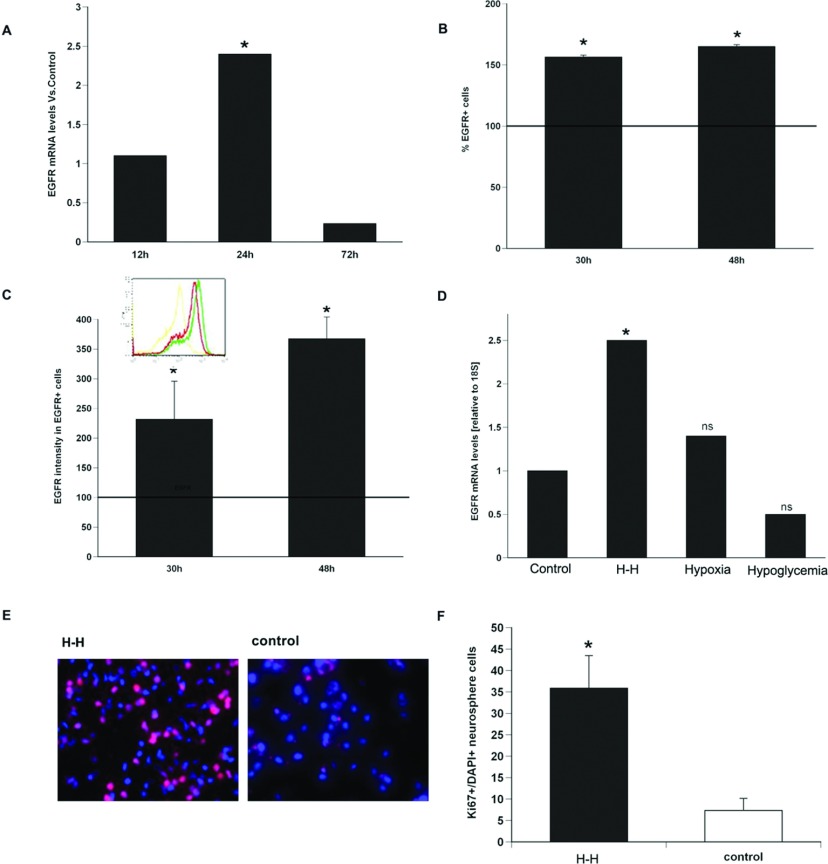
EGF-R induction and cell division can be recapitulated *in vitro* in the absence of a brain cell niche (**A**) Q-PCR using primers specific for EGF-R and normalized to expression of 18S at 12, 24 and 72 h after *in vitro* H**–**H on rat NPs. (**B**) Quantification of the EGF-R expressing cells and (**C**) EGF-R intensity within NPs at 30 and 48 h after *in vitro* H**–**H. (**D**) EGF-R mRNA at 24 h after exposure of NPs to only hypoxia (2% O_2_) or only ischemia (3 mM glucose) or a combination of both. Solid line indicates EGF-R levels thee in untreated control NPs. Values are average±S.E.M. from three independent experiment with *n*=6 animals per experiment.*=*P*<0.05 by REST (relative expression software tool). (**E**) NP were exposed to H–H, allowed to recover for 30 h and then labelled for Ki67 (red) and counterstained with DAPI (blue). (**F**) Quantification of % Ki67/total cells. Values represent mean±S.E.M.*P<0.5 by Student's *t* test.

In a previous study, we demonstrated that EGF-R expression increases on a population of primitive NPs in the SVZ that express the Lewis antigen (Alagappan et al., 2009). To determine whether the increase in the EGF-R observed after neonatal H–I was a direct consequence of reduced oxygen and reduced glucose, we evaluated the responses of NPs to hypoxia, hypoglycemia or the combination *in vitro*. Primary neurospheres from Wistar rat pups were subjected to low oxygen (2%) or low glucose (3 mM) or the combination for 4 h to simulate H–I *in vitro.* The cells were returned to standard neurosphere culture conditions (17.5 mM glucose and 20% (v/v) O_2_) for 12, 24 and 72 h. At these time points, EGF-R mRNA levels were 1.2, 2.4 and 0.3-fold compared with control spheres. EGF-R mRNA levels were significantly (*P*<0.05) elevated compared with control spheres at the 24 h time point (Figure 1A).

To analyse EGF-R protein levels, rat neurospheres were subjected to combined hypoxia–hypoglycemia (H–H) and then allowed to recover for 30 and 48 h after which EGF-R expression was evaluated by flow cytometry. By 30 h there were 56% more NPs expressing EGF-R from neurospheres subjected to H–H compared with control neurospheres, and by 48 h there were 65% more EGF-R+ NPs (at 30 h after H–H 76.73%±1.41 EGF-R+cells versus 20.3±2.29 for controls. At 48 h after H–H 85.23%±1.59 EGF-R+cells versus 21.8±2.2 for controls NS; mean±S.E.M., *n*=3 independent experiments with six animals per experiment) (Figure 1B). To determine whether the levels of receptor expression per cell differed between the cells exposed to H–H versus controls we measured the mean geometric fluorescence for EGF-R on the EGF-R NPs at 30 and 48 h recovery from H–H. These analyses found that the amount of EGF-R expressed per cell increased by 2-fold and 2.9-fold (*P*<0.05) compared with controls. At 30 and 48 h, respectively, after H–H measured fluorescence levels were 400±38.69 and 576±78.56 versus control levels of 194±51.09; mean±S.E.M, in a.u. (arbitrary units), *n*=3 independent experiments with six animals per experiment (Figure 1C).

To determine whether either hypoxia or hypoglycemia alone was sufficient to increase the expression of the EGF-R, rat neurospheres were exposed to either hypoxia (2% oxygen/5%CO_2_/93% N_2_) or hypoglycemia (3 mM glucose) for 4 h following which the cells were returned to standard neurosphere culture conditions for 24 h. This time point was selected as the EGF-R mRNA was maximally induced by 24 h. EGF-R mRNA levels were quantified using Q-PCR with EGF-R specific primers. Neither hypoxia nor ischemia independently induced EGF-R mRNA. Levels of EGF-R mRNA increased 1.4-fold (*P*>0.05) with only hypoxia alone and were reduced by 0.5-fold with hypoglycemia alone (*P*>0.05) versus control (ns). By contrast, combining *in vitro* hypoxia with hypoglycemia increased EGF-R mRNA levels by 2.5-fold (*P*<0.05), (Figure 1D).

As EGF is a potent mitogen for NPs, we asked whether H-H induced levels of the EGF-R would increase their growth rate. Rat SVZ NPs were subjected to H-H, allowed us to recover for 30 h and then immunostained for Ki67 together with the nuclear stain DAPI. When the percentage of Ki67^+^/DAPI^+^ cells was compared between H-H exposed NPs versus control NPs, there was a 5-fold increase in Ki67^+^ cells following this stress compared with control NPs that were not subjected to H–H (Figures 1E and 1F).

### Zinc finger transcription factor Egr-1 increases after H–H and induces EGF-R production in SVZ NPs

Multiple transcription factors are induced in response to H–I stress in cells including Egr-1, HIF-1α, Ras, NF-κB, Src, p53 and AP-1 (Wang and Semenza, 1993; Graeber et al., 1994; Koong et al., 1994; Muller et al., 1997; Yan et al., 1999). However, it is presently unknown which transcription factors regulate EGF-R expression after brain injury. As the EGF-R promoter sequence has an Egr-1 binding consensus sequence whereas there is no reported HIF-1 responsive element upstream of the EGF-R gene coding region (Nishi et al., 2002), and studies reported that Egr-1 can bind DNA and activate EGF-R transcription in tumour cells (Nishi et al., 2002) we tested the hypothesis that Egr-1 was necessary for induced EGF-R expression within SVZ NPs in response to H–H stress.

**Figure 2:**
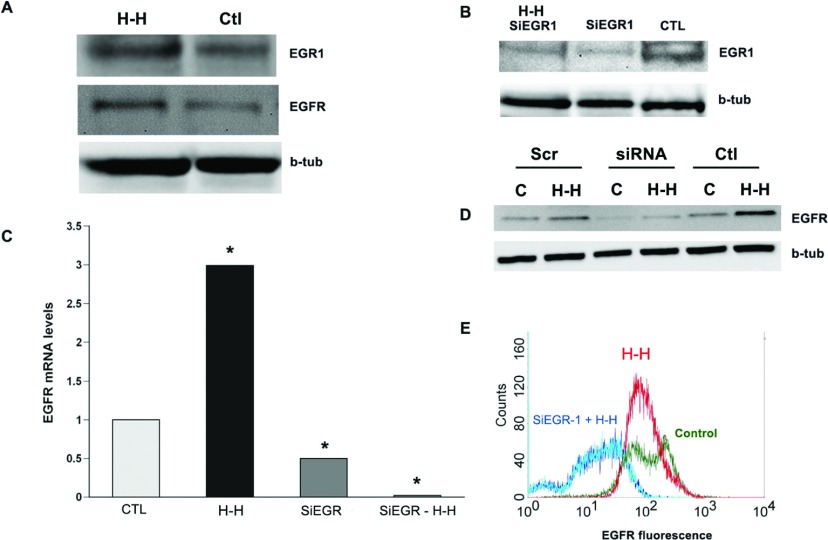
The increase in EGF-R requires Egr-1 (**A**) Rat NPs were subjected to *in vitro* H–H (3 mM glucose media and 2% O_2_ for 4 h) after which the spheres were lysed and analysed for the presence of Egr-1 accumulation and EGF-R. (**B**) Rat NPs were transfected with SiRNAs against Egr-1 and subjected to H–H and analysed for the expression of Egr-1. Control NPs were mock transfected and not subjected to H–I. β-tubulin was used as a loading control. (**C**) Q-PCR using primers specific for EGF-R normalized to 18S expression in rat NPs subjected to *in vitro* H–I with or without the transfection of SiRNAs to Egr-1. Control NPs were mock transfected and not subjected to H–I. (**D**) Cell lysates from spheres subjected to H–I (*lanes b*,*d*,*f*) and control spheres (*lanes a*,*c*,*e*) were analyed for EGF-R expression. Cells were transfected with SiRNAs against Egr-1 (*lanes c*,*d*) or random scrambled oligonucleotide sequences (*lanes a*,*b*). (**E**) Quantification of the EGF-R intensity by flow cytometry (in a.u.) within EGF-R expressing cells transfected with SiRNA to Egr-1 and untransfected NPs subjected to H–I *in vitro*. *n*=3 independent experiments with six animals per experiment.*=*P*<0.05 by REST (Pfaffl et al., 2002).

Rat SVZ NPs were subjected to H–H and evaluated immediately following this stress for Egr-1 accumulation by Western blot. Confirming our hypothesis, Egr-1 accumulated (as did EGF-R) within NP cell lysates subjected to H–H compared with control cell lysates (Figure 2A). To determine whether Egr-1 is necessary for EGF-R induction following H–H, NPs were transfected with ON-TARGET plus SiRNA against Egr-1 (SiEgr-1) (Dharmacon, Cat# L-040286-00) or with control ON-TARGET *plus* Non-targeting Pool (Dharmacon, Cat # D-001810-10-05) using the Amaxa transfection system. Transfected NPs were subjected to H-H and then allowed to recover for 30 h. NPs that were mock transfected were used as additional controls. Both basal and stress-induced Egr-1 accumulation was suppressed in SiEgr-1 transfected cells after H–H (Figure 2B). We measured levels of EGF-R mRNA in these transfected cells and found that H–H induced a 3-fold increase in EGF-R mRNA levels in control transfected NPs versus NPs maintained under normoxic-normoglycemic conditions. However, there was a 2-fold reduction in basal EGF-R expression in the absence of H–H in NPs transfected with SiEGR-1 compared with mock-transfected control spheres (Figure 2C). Supporting our hypothesis, EGF-R was reduced 25-fold in NPs transfected with SiRNAs to Egr-1 and subjected to H–H compared with NPs transfected with SiRNAs to Egr-1 but not subjected to H–H. Western blots for EGF-R demonstrated that EGF-R in SiEgr-1 transfected cells was not induced by H–H compared with controls (Figures 2C and 2D).

Next cell surface expression of EGF-R was evaluated after H–H in control and siEgr-1 transfected NP using flow cytometry. NPs transfected with SiRNAs to Egr-1 and subjected to H–H showed a significant leftward shift in the flow analysis due to a decrease in EGF-R expression per cell as well as reduced numbers of cells expressing detectable levels of EGF-R. As observed with EGF-R mRNA levels, suppressing Egr-1 using SiRNAs reduced basal cell surface expression of EGF-R as shown by a decrease in the peak amplitude (Figure 2E).

### Egr-1 does not accumulate in astrocytes in response to hypoxic–hypoglycemic stress

**Figure 3:**
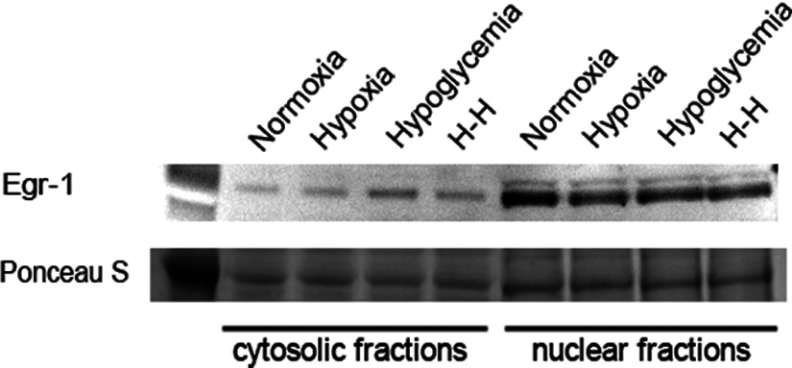
Egr-1 does not accumulate in astrocytes in response to hypoxic–hypoglycemic stress Highly enriched cultures of neocortical astrocytes were generated from P1-2 rat pup mixed glial cell cultures and then maintained in a hormone-supplemented medium for 2 days to promote differentiation. The astrocytes were exposed to 4 h of either hypoxia alone (2% O_2_), hypoglycemia alone (3 mM glucose) or H–H (3 mM glucose media and 2% O_2_ for 4 h) and then returned to standard culture conditions for 24 h whereupon the cells were subjected to subcellular fractionation and analysed for the presence of Egr-1 accumulation. Data are representative of three independent experiments.

Neural stem cells within the SVZ have been characterized as astrocyte-like cells (Doetsch et al., 1999). Therefore it was of interest to establish whether the increase in Egr-1 in response to H–H is a general response of astrocytes and astrocyte-like cells. Highly enriched cultures of neocortical astrocytes were generated from P1–2 rat pup mixed glial cell cultures (Levison and McCarthy, 1991) and then maintained in a biochemically defined medium for 2 days to promote their differentiation. The astrocytes were exposed to 4 h of either hypoxia alone, hypoglycemia alone or H–H and then returned to standard culture conditions for 24 h whereupon the cells were subjected to subcellular fractionation. Contrary to our expectations, Egr-1 did not accumulate in either the cytoplasm or the nuclei of astrocytes exposed to these stresses (Figure 3).

### Egr-1 accumulates within nucleus of SVZ NPs following injury

**Figure 4:**
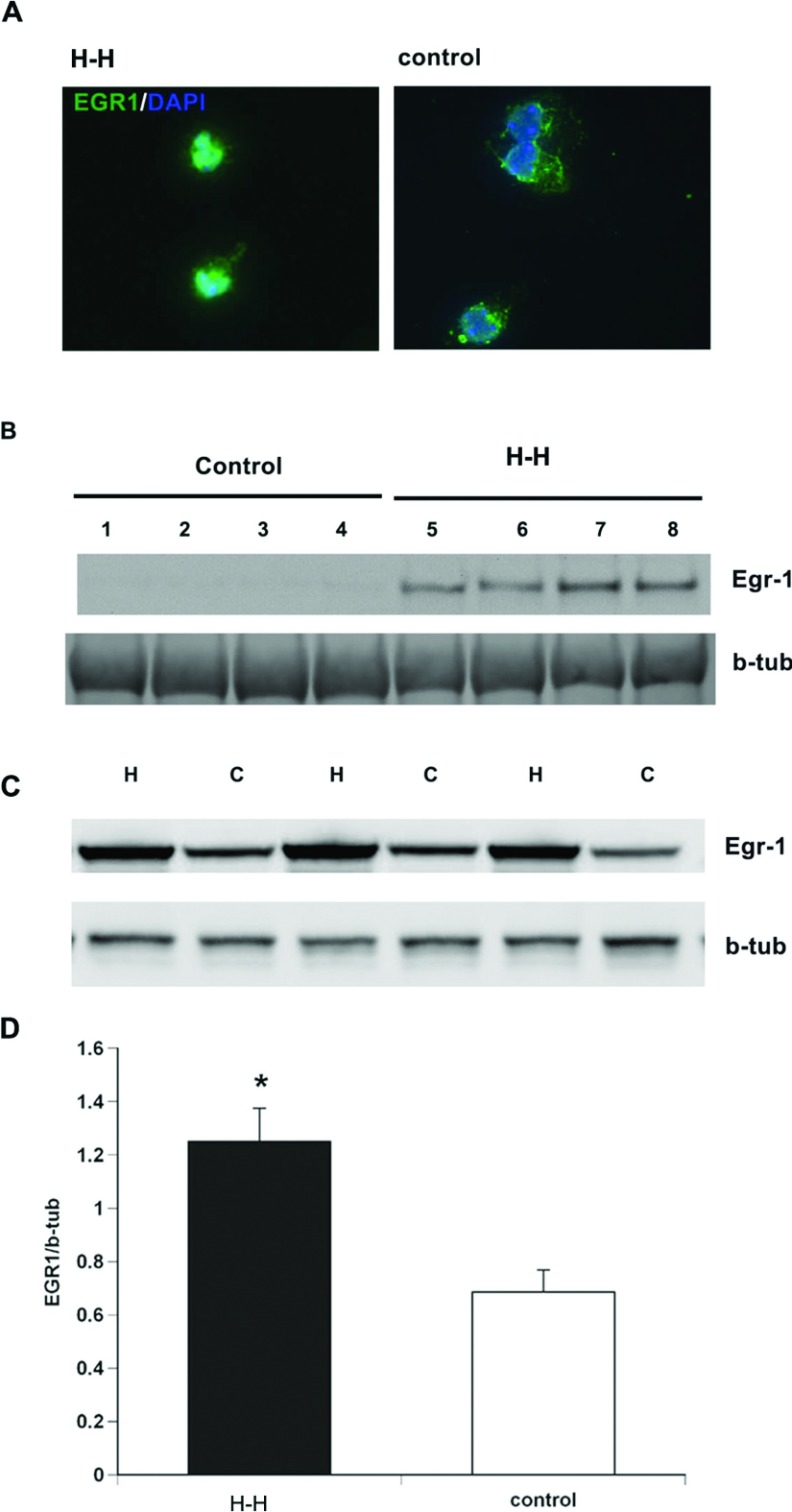
Egr-1 accumulates in the nucleus following H–I and binds the EGF-R promoter (**A**) Rat neurospheres were subjected to H–H and stained for Egr-1 (green) and counterstained with DAPI (blue). (**B**) Nuclear extracts from neurospheres subjected to H–H and control neurospheres and probed with antibodies specific to Egr-1. (**C**) Egr-1 levels in NPs subjected to hypoxia alone compared with control NPs. (**D**) Densitometric quantification of Western blots. Values represent mean±S.E.M.*P<0.05 by Student's *t* test.

Demonstrating that a transcription factor accumulates within a cell during recovery from a stressor is insufficient to conclude that it is acting as a transcription factor to promote EGF-R synthesis within SVZ NPs following injury. Therefore to more firmly establish that Egr-1 mediates the transcription of EGF-R within SVZ NPs, we evaluated Egr-1 levels within the nuclei of NPs during recovery from H–H stress. First, rat SVZ NPs were subjected to H–H and then allowed to recover for 30 h, following which the cells were stained for Egr-1 together with the nuclear stain DAPI. Epifluorescence microscopic analyses established that Egr-1 accumulated in the nucleus during recovery from H–H (Figure 4A). To quantify nuclear Egr-1, extracts from NP nuclei that had been subjected to H–H were compared to naïve control nuclear extracts by Western blotting. Consistent with the immunofluorescence analysis, Egr-1 proteins were two orders of magnitude increased in the nuclei of cells subjected to H–H compared with controls (Figure 4B). Additional studies were performed to establish whether hypoxia alone was sufficient to increase levels of Egr-1. These studies showed that indeed, hypoxia alone increased levels of Egr-1 by 2-fold (*P*<0.05) (Figures 4C and 4D).

The EGF-R promoter sequence has an Egr-1 binding consensus sequence. To establish whether there is elevated DNA-binding of Egr-1 to the Egr-1 consensus sequence on EGF-R promoter sequence following H-H, we subjected NPs to H-H, allowed the cells to recover for 30 h, and then performed an electrophoretic mobility shift assay using an oligomer derived from the EGF-R promoter. These studies revealed a shift in the mobility of the ^32^P-labeled EGF-R promoter sequence in NPs subjected to H–H compared with controls (results not shown). Efforts to obtain a mobility shift using antibodies against Egr-1 were regrettably unsuccessful despite multiple trials (Alagappan, 2008).

**Figure 5:**
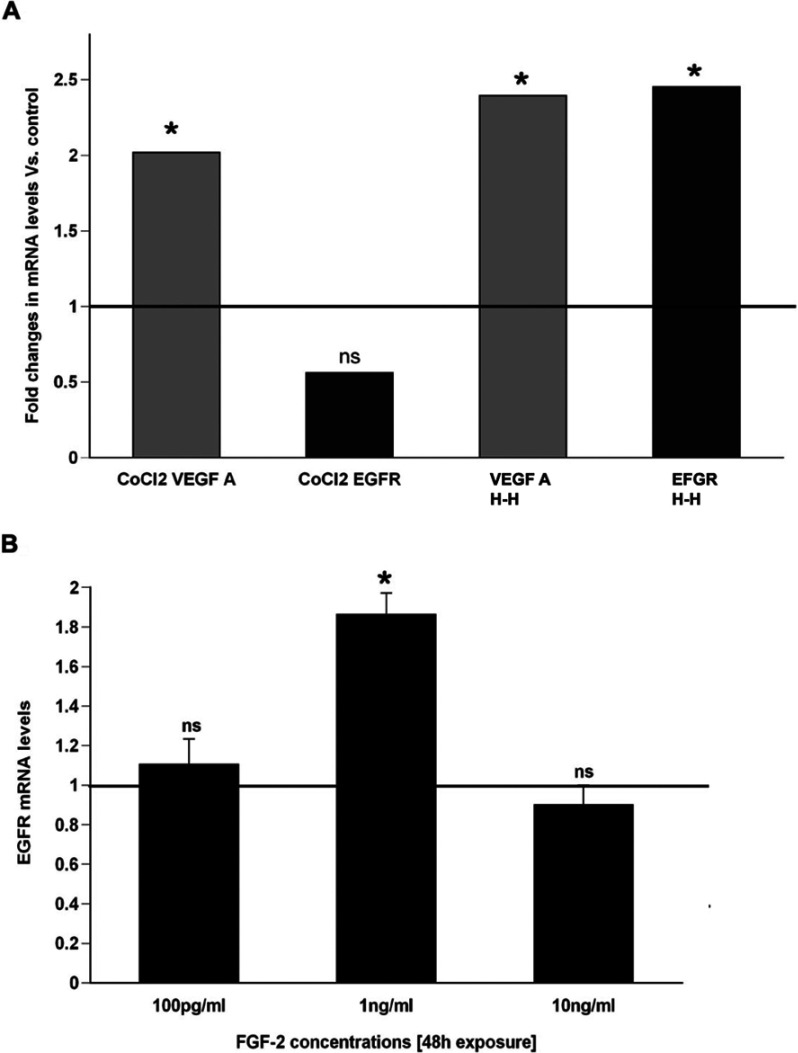
FGF weakly induces EGF-R (**A**) 300 mM CoCl_2_ was used to induce HIF-1 and the levels of EGF-R mRNA were analysed using Q-PCR. VEGF mRNA was used as a positive control for HIF-1 induced transcription. (**B**) Primary neurospheres were generated in EGF (2 ng/ml) supplemented media and exposed to FGF-2 for 48 h following which the spheres were collected and the EGF-R levels were analysed by Q-PCR and normalized to 18S expression levels. Solid line indicates EGF-R levels in untreated control spheres. Values are average±S.E.M. from three independent experiment with *n*=6 animals per experiment.*P<0.05 by REST.

An analysis of the EGF-R promoter did not reveal a canonical HIF response element; yet, HIF is induced by H–H. Therefore to determine whether HIF might regulate EGF-R mRNA synthesis, we added cobalt chloride, a chemical inducer of HIF, to the neurosphere culture medium and evaluated the levels of EGF-R mRNA transcripts. Whereas H–H induced a 2.5-fold induction of EGF-R mRNA, cobalt chloride did not significantly induce EGF-R expression compared with controls. By contrast, VEGF-A mRNA, a growth factor well known to be regulated by HIF, was induced over 2-fold by cobalt chloride treatment and by H–H (Figure 5A).

FGF-2 also increases after H–I and FGF-2 is known during development to increase the expression of EGF-R in SVZ cells (Lillien and Raphael, 2000); therefore we asked whether FGF-2 would increase EGF-R mRNA in SVZ cells. FGF-2 did increase EGF-R mRNA; however it did so only modestly (1.9-fold versus control) and it exerted this effect only at 1 ng/ml with no effect at either 10-fold higher or 10-fold lower concentrations (Figure 5B).

## DISCUSSION

In an earlier study, we used flow cytometry on SVZ cells isolated acutely after neonatal H–I and showed that EGF-R expression increased significantly on primitive NPs of the SVZ during recovery from neonatal H–I. Furthermore, we showed that pharmacologically inhibiting the EGF-R reduced the expansion of the NPs that normally occurs. We also showed that overexpressing a constitutively active EGF-R was sufficient to increase the proportion of cycling NPs (Alagappan et al., 2009). Here we tested the hypothesis that the induction of EGF-R on SVZ NPs following stress requires Egr-1 accumulation. We show that: (1) the *in vivo* increase in EGF-R expression levels can be recapitulated *in vitro* by exposing neonatal SVZ NPs to hypoxia and hypoglycemia simultaneously, but not separately; (2) exposing SVZ NPs to hypoxia and hypoglycemia simultaneously increases the percentage of cycling cells; (3) Egr-1 expression increases following *in-vitro* hypoxia and hypoglycemia and Egr-1 predominantly accumulates in the nucleus; (4) Egr-1 does not accumulate in neonatal neocortical astrocytes exposed to *in vitro* hypoglycemia and hypoxia; (5) decreasing Egr-1 expression in NPs using SiRNAs specific for Egr-1 decreases both basal as well as the stress-induced increase in EGF-R expression; (6) HIF-1 accumulation does not appear to contribute to EGF-R expression after hypoxia and hypoglycemia in NPs and (7) FGF-2 only modestly induces EGF-R in newborn SVZ NPs.

### Multiple transcription factors may induce EGF-R expression post injury

Several DNA-binding factors act as transcriptional regulators of EGF-R expression such as WT1, TCC, AP-1, AP-2, SP-1 and p53. Following injury or stress, such as hypoxia, many of these transcription factors are induced, but the specific transactivating factor (s) responsible for the hypoxia-induced increase in EGF-R on NPs has not been elucidated. Studies by Nishi et al. (2002) showed that Egr-1, a transcriptional regulator of ‘inducible’ genes associated with stress responses, was induced in response to hypoxia and that Egr-1 could alter the basal transcriptional activity of EGF-R in several somatic tissue cancer cell lines (U-2OS, Saos-1, KB and HeLa). Overexpressing Egr-1 in these cancer cell lines activated the basal transcriptional activity of EGF-R promoter by direct DNA-binding, which enhanced EGF-R expression. Using electrophoretic mobility shift assays, the investigators demonstrated that Egr-1 binds to EGF-R promoter regions and that an antisense oligonucleotide for Egr-1 reduced EGF-R expression following hypoxic stress (Nishi et al., 2002). On the basis of their studies, we hypothesized that Egr-1 was a crucial regulator of EGF-R expression within SVZ NPs observed following H–I and the studies reported here fully support that hypothesis.

### FGF-2 is a relatively minor regulator of EGF-R expression whereas Egr-1 appears to be necessary for both basal and induced EGF-R expression on NPs following H–H

Several groups have shown that FGFs regulate embryonic cortical and striatal precursor cell EGF-R acquisition during development (Ciccolini and Svendsen, 1998; Lillien and Raphael, 2000). In previously published work, our laboratory showed that several FGF ligand genes are induced during recovery from neonatal H–I (Felling et al., 2006). Therefore we asked whether FGF-2 could recapitulate the increase in EGF-R expression on SVZ cells. However, contradicting the prediction that FGF-2 was regulating EGF-R expression, FGF-2 only modestly increased EGF-R and exerted this effect within a very narrow does effect range (Figure 5). This is perhaps not surprising since these SVZ NPs *in vitro* already express the EGF-R.

Egr-1 is known to be induced by the Erk signal transduction pathway and it has been shown to be essential for mammary epithelial cells to transverse a threshold to enter S-Phase of the cell cycle (Zwang et al., 2011). Here we document a new role for Egr-1 in regulating cell proliferation. We found that Egr-1 was necessary for both basal and induced expression of EGF-R. When NPs from the SVZ were transfected with SiRNAs against Egr-1, basal expression of EGF-R decreased 2-fold and the increase in EGF-R after H–H was reduced to almost undetectable levels (Figure 2).

### Hypoxia and hypoglycemia coordinately induces Egr-1 in NPs but not in neocortical astrocytes

The somatic neural stem cells within the SVZ have been characterized as astrocyte-like cells in that they express several proteins that have been previously been regarded as characteristic of astrocytes, such as GFAP, GLAST and Musashi-1 and they extend a process to blood vessels (Doetsch et al., 1999). Therefore it was of interest to establish whether the increase in Egr-1 in response to H–H is a general response of astrocytes and astrocyte-like cells. Moreover, astrocytes acquire EGF-R after injury, which promotes their proliferation in response to elevated levels of TGFalpha, heparin-binding EGF and other EGF-R ligands (Xian and Zhou, 1999). However, H–H did not induce Egr-1 in neocortical astrocytes, contrary to our predictions. In retrospect, this result is not surprising, since neural stem cells are distinct from astrocytes. Astrocytes are predominantly post-mitotic cells and they perform numerous functions to maintain metabolic and ionic homoeostasis in the brain, whereas stem cells are undifferentiated, cycling cells. We recently demonstrated that hypoxia increased the expression of astrocytic glutamine synthetase and the monocarboxylate transporter in astrocytes, which correlated with an increase in their buffering capability for glutamate and their ability to protect neurons from an excitotoxic insult (Bain et al., 2010) These are not functions that a neural stem cell would be expected to perform. Thus, our current data support a model that can be tested in future experiments, where different transcription factors regulate the induction of those molecules that enhance an astrocyte's capacity to protect neurons from injury. Other transcription factors, like Egr-1 will regulate those molecules that are required by these precursors to generate new cells.

### Egr-1 stabilization may contribute to Egr-1 accumulation in the nucleus to promote EGF-R transcription after H–I

Egr-1 is a short-lived protein with a half-life of ~2 h. Bae et al. (2002) reported that Egr-1 levels are regulated via an ubiquitin-dependent proteosomal degradation pathway by specific interaction of Egr-1 with the proteasome component C8 (Bae et al., 2002). Thus, polyubiquitination of Egr-1 serves as a degradation signal and under standard conditions Egr-1 protein is constantly cleared from the cell by cytosolic proteasomes. However, following hypoglycemia and hypoxia, Egr-1 is stabilized and escapes degradation, which we have confirmed in our studies. As Egr-1 accumulates it traffics to the nucleus to promote or repress specific genes. We attempted to evaluate cytoplasmic versus nuclear localization of Egr-1 after neonatal H-I by performing immunofluorescence for Egr-1 at 4 h of recovery from injury using the Vannucci model of neonatal H–I. We evaluated several commercially available antibodies to Egr-1. Unfortunately, the Egr-1 staining intensity was very low in the SVZ and we did not observe any significant increase in levels or a change in subcellular localization at the 4 h time-point of recovery from H–I. Therefore, while our *in vitro* studies clearly demonstrate an important role for Egr-1 in regulating NP proliferation in response to the combined insult of hypoxia and hypoglycemia, additional studies will be necessary to confirm the importance of this transcription factor for the proliferative response to brain injury *in vivo*.

### Egr-1 may require co-activators to bind EGF-R promoter sequences to enhance EGF-R transcription following H–I

Although Egr-1 has been implicated in the cellular response to hypoxia with enhanced expression of the EGF-R, we failed to see EGF-R induction in NPs when exposed to hypoxia or hypoglycemia alone. Exposure to hypoxia and hypoglycemia simultaneously was required for EGF-R induction. These results suggest that there are additional factors that are induced in response to hypoglycemia that might bind to Egr-1 or bind to EGF-R promoter sequences to act as transcriptional co-activators to enhance EGF-R transcription following hypoxia and ischemia.

Ischemia increases the levels of glucose transporters, such as GLUT1 and GLUT3, in several tissues including heart and brain (Calvert et al., 2006; Davey et al., 2007). Interestingly, ischemia increases the levels of GLUT-1 in the brain via Sp1, a transcription factor that has specific binding sequences within the GLUT-1 gene (Santalucia et al., 1999) as well as within the EGF-R promoter (Nishi et al., 2002). Studies in human hepatocellular liver carcinoma cell line, HepG2, cells show that hypoxia does not induce Sp1 but instead induces Egr-1 (Bae et al., 1999). The above data lead us to surmise that Sp1 may be induced following hypoglycemia within NPs and then that SP-1 acts either directly by binding Egr-1 or indirectly by binding EGF-R promoter sequences to induce EGF-R expression following exposure to hypoxia and hypoglycemia.

### Conclusion

In conclusion, our studies demonstrate that Egr-1 is a critical regulator of EGF-R expression, which promotes the regenerative response of the stem/progenitor cell pool within the SVZ during recovery from neonatal H-I brain injury. We show that Egr-1 accumulates within the nucleus subsequent to a hypoxic-ischemic insult and that knocking down Egr-1 is sufficient to dramatically reduce both basal and induced EGF-R expression. These data provide new insights into mechanisms that regulate CNS regeneration and they suggest strategies for amplifying the numbers of stem cells for CNS regeneration.
